# Involvement of Aryl Hydrocarbon Receptor in Longevity and Healthspan: Insights from Humans, Mice, and *C. elegans*

**DOI:** 10.3390/ijms25189943

**Published:** 2024-09-14

**Authors:** Eva Serna, David Verdú, Alicia Valls, Ángel Belenguer-Varea, Francisco José Tarazona-Santabalbina, Consuelo Borrás, José Viña

**Affiliations:** 1 Department of Physiology, School of Medicine, University of Valencia, CIBERFES, INCLIVA, 46010 Valencia, Spain; david.verdu@uv.es (D.V.); alicia.valls@uv.es (A.V.); consuelo.borras@uv.es (C.B.); jose.vina@uv.es (J.V.); 2 MODULAhR Group, University of Valencia, 46010 Valencia, Spain; 3 Division of Geriatrics, Hospital Universitario de La Ribera, 46600 Valencia, Spain; abelenguervarea@gmail.com (Á.B.-V.); fjtarazonas@gmail.com (F.J.T.-S.)

**Keywords:** aryl hydrocarbon receptor, lifespan, vitality, pomegranate extract

## Abstract

In previous studies, using transcriptomic analysis, we observed higher levels of aryl hydrocarbon receptor (AHR) gene expression in the peripheral blood cells of centenarians compared to octogenarians. This suggests the potential significance of this receptor in maintaining physiological balance and promoting healthy aging, possibly linked to its critical role in detoxifying xenobiotics. In our current study, we confirmed that AHR expression is indeed higher in centenarians. We employed *C. elegans* as a model known for its suitability in longevity studies to explore whether the AHR pathway has a significant impact on lifespan and healthspan. Our survival assays revealed that two different mutants of AHR-1 exhibited lower longevity. Additionally, we used a mouse model to examine whether supplementation with pomegranate extract modulates the expression of AHR pathway genes in the liver. Furthermore, we studied a nutritional strategy based on pomegranate extract administration to investigate its potential modulation of life- and healthspan in worms.

## 1. Introduction

As the global population ages at an unprecedented rate, the study of longevity and healthy aging has garnered increasing attention as an essential societal priority. According to the World Health Organization (WHO), by 2050, the number of people aged 60 years and older is expected to more than double, reaching approximately 2 billion individuals worldwide. This demographic shift underscores the pressing need to understand factors that contribute to a long and healthy life, not only for individual well-being but also for the socioeconomic stability of countries.

In this context, centenarians emerge as an exceptional study group, providing a unique opportunity to understand the biological mechanisms underlying exceptional aging. In previous preliminary studies, we revealed higher levels of aryl hydrocarbon receptor gene expression in the peripheral blood cells of centenarians compared to octogenarians and a similar expression with young people [1]. The aryl hydrocarbon receptor (AHR) is a homeostatic sensor crucial for detoxifying xenobiotics [2,3]. Furthermore, AHR is involved in a variety of physiological functions, including cell regeneration, immune response modulation, intestinal homeostasis maintenance, and cell proliferation regulation [1,4]. Previous research showed that the absence or inadequate levels of AHR can lead to premature aging, the dysregulation of hematopoietic stem cells, the disruption of glucose and lipid homeostasis, increased inflammation, and neurological decline [5,6,7,8,9].

This highlights the importance of this receptor in maintaining homeostasis and promoting healthy aging. This is attributed, among other reasons, to its fundamental role in activating cytochrome P450 in detoxification response [10]. It is this capability to neutralize environmental toxins that could partly contribute to the observed longevity and healthspan in centenarians. Chronic exposure to environmental contaminants can exert considerable pressure on the body’s detoxification systems, exacerbating oxidative stress and triggering inflammatory processes that accelerate aging [11]. However, AHR’s ability to modulate the expression of detoxifying enzymes such as cytochrome P450 confers a unique adaptive advantage to long-lived individuals by allowing them to more effectively confront these environmental challenges.

Nutritional strategies, such as incorporating pomegranate (*Punica granatum* L.), have been helpful in modulating both life- and healthspan [12,13,14]. Pomegranate (*Punica granatum* L.) is a polyphenol-rich food containing flavonols, anthocyanins, and tannins. Pomegranate extract (PE) presents beneficial properties for health, such as antioxidant activity and anti-cancer, anti-inflammatory, and anti-aging effects [15]. Furthermore, pomegranate and its constituents have shown protective effects against natural toxins and chemical toxicants. Their protective effects are associated with different mechanisms such as the prevention of oxidative stress; reduction in inflammatory mediators including tumor necrosis factor-alpha (TNF-α), interleukin-6 (IL-6), cyclooxygenase-2 (COX-2), and nuclear factor ĸB (NF-ĸB); and the modulation of apoptosis and mitogen-activated protein kinase (MAPK) signaling pathways [16]. Urolithin A is a major microbial metabolite derived from polyphenolics of pomegranate fruits, and it shows the same favorable effects such as anti-inflammatory, antioxidative, and anti-aging activities [12,17]. Furthermore, urolithin A increases gut barrier integrity through the activation of AHR- and nuclear factor erythroid 2-related factor 2 (Nrf2)-dependent pathways [18,19].

The aim of this study was to investigate the AHR gene expression pathway in a cohort comprising centenarians and their septuagenarian offspring and comparing them with septuagenarians who are non-centenarian offspring. Additionally, we explored the role of AHR in regulating lifespan in *C. elegans*. We further investigated the effects of pomegranate extract (PE) on the regulation of the AHR pathway in the liver of mice, as well as its impact on both longevity and vitality in *C. elegans*. Our findings suggest that the beneficial effects of PE on longevity may be mediated by the AHR-1 receptor.

## 2. Results

### 2.1. Human Study: AHR Canonical Pathway Is Up-Regulated in Centenarians and Their Offspring

We conducted a validation cohort study to corroborate our previous findings [1] wherein we observed that the AHR pathway was overexpressed in centenarians compared to ordinary septuagenarians. Our present data revealed that centenarian offspring exhibit a similar expression pattern to centenarians in almost all genes involved in this pathway. Thus, centenarians and septuagenarians who are centenarian offspring are similar but different from non-centenarian offspring. We measured the expression levels of the following genes: *AHR*, *ARNT*, *CYP1B1*, and *NFR2* (*NFE2L2*) (Table 1). The difference between expression in centenarians and ordinary septuagenarians was statistically significant (*p* < 0.05). It must be emphasized that the groups of centenarian offspring are not descendants of the centenarians included in this cohort.

**Figure 1:**
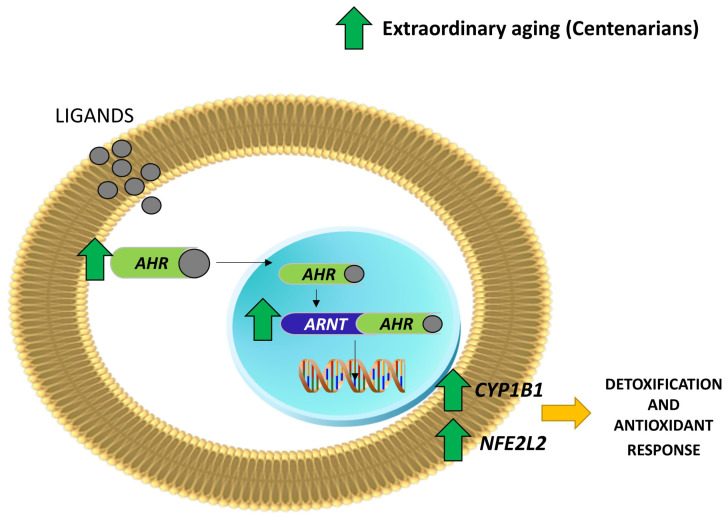
Graphical summary of AHR canonical pathway that is up-regulated in centenarians.

This indicates that the AHR pathway exhibits similarity between centenarians and offspring but significant dissimilarity between centenarians and septuagenarians. Thus, the AHR pathway is overexpressed in extraordinary aging (centenarians) (see Figure 1).

**Figure 2:**
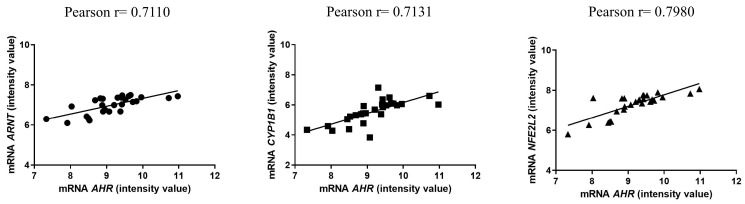
Correlation of all genes involved in the AHR canonical pathway. Circle symbols represent the correlation between *AHR* and *ARNT*, square symbols represent the correlation between *AHR* and *CYP1B1,* and triangle symbols represent the correlation between *AHR* and *NFE2L2*.

In Figure 2, we depict the logistic regression analysis of the gene expression levels of the following AHR pathway genes: *AHR* and *ARNT*, *AHR* and *CYP1B1*, and *AHR* and *NFE2L2* (*NRF2*). All analyzed data exhibit a significant positive correlation (*p* < 0.0001), indicating that this pathway is activated in centenarians and not in septuagenarians. This activation suggests enhanced homeostasis and improved detoxification and antioxidant response capability within this group.

### 2.2. C. elegans Study: Involvement of AHR-1 in Lifespan

To assess the role of the AHR-1 receptor (containing 602 amino acids) in lifespan, survival assays were repeated with two different mutants available in the Caenorhabditis Genetics Center (CGC) repository: CZ2485 ahr-1 (ju145) containing 302 amino acids and ZG24 ahr-1 (ia3) containing 434 amino acids. The ahr-1(ju145) mutant contains a nonsense mutation generating a shorter coding protein (302 amino acids versus 602 amino acids) not presenting modifications in the known functional domains, in the binding sequence to DNA (HLH domain), or in the sequence with the function of sensing molecules (PAS domain). On the other hand, the ahr-1(ia3) mutant presents a deletion of 168 amino acids, losing part of the PAS domain.

**Figure 3:**
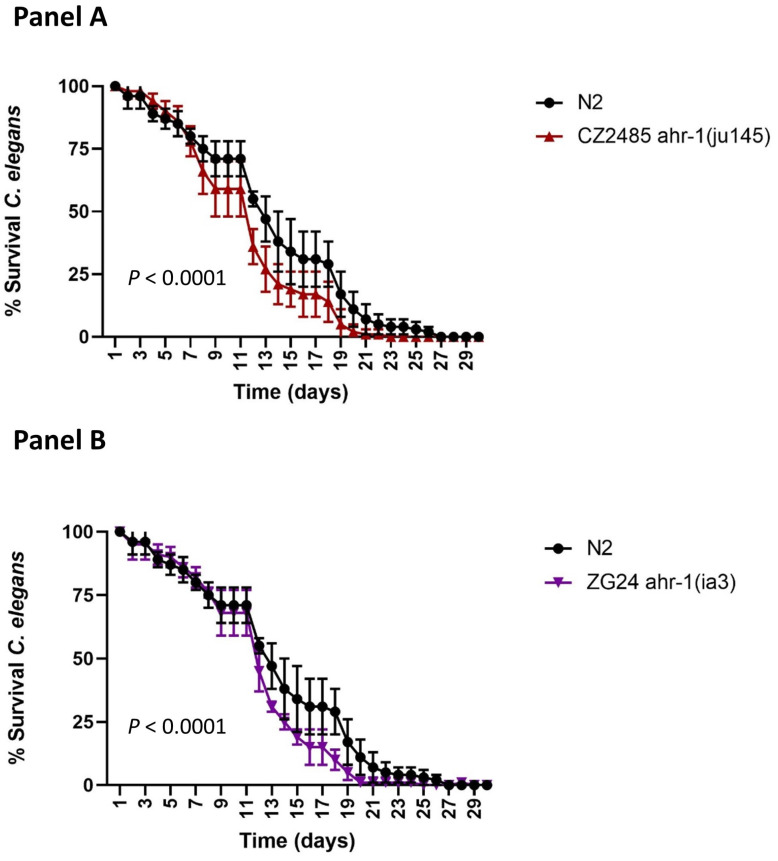
Survival curves of *C. elegans* mutants ahr-1 (ju145) (**Panel A**) and ahr-1 (ia3), (**Panel B**) compared with wild-type strain (N2). Data correspond to *n* = 200 worms/condition. *p* < 0.0001.

Previous publications showed a higher survival of the ahr-1(ju145) mutant in *C. elegans* [20]. However, in our hands, as is shown in Figure 3, none of our mutants analyzed showed a positive effect on lifespan compared to the wild-type strain (N2). In fact, a decrease in survival in both mutants was observed. The reason for this discrepancy is explained in the Discussion Section.

### 2.3. Nutritional Intervention with Pomegranate Extract on AHR Pathway to Promote Longevity in C. elegans

We tested the effect of pomegranate extract on the AHR pathway and on *C. elegans* longevity. Initially, the doses of 10, 5, and 1 mg/mL were evaluated, observing no positive effect on the lifespan of the worms. Subsequently, lower doses of the extract (0.005, 0.01, 0.05, 0.1, and 0.5 mg/mL) were analyzed. Worms fed with doses of pomegranate extract of 0.05, 0.1, and 0.5 mg/mL displayed significantly higher survival than worms fed under control conditions (Table 2). The doses of 0.05 and 0.1 mg/mL were optimal, without significant differences between them. In addition, these doses presented an increase in the half-life (day on which survival is 50%) of six days (0.05 mg/mL) and two days (0.1 mg/mL) compared with the vehicle (non-supplemented group). They also showed a prolongation of maximal lifespan (the day on which mortality is 100%) compared to vehicle. The 0.5 mg/mL dose was also able to increase the half-life of the worm, with a difference of 2 days compared to the control (Table 2).

**Figure 4:**
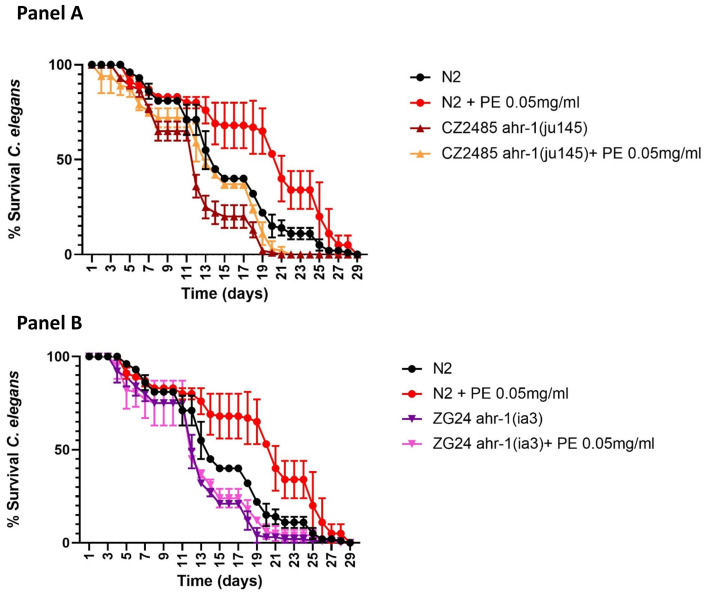
Effect of PE 0.05 mg/mL on lifespan in mutants *C. elegans* ahr-1 (ju145) (**Panel A**) and ahr-1 (ia3) (**Panel B**) in comparison with wild-type strain (N2). Data corresponded to *n* = 100 worms/condition. PE: pomegranate extract.

We proceeded to evaluate the role of the AHR-1 receptor in the effect of PE on survival. For this, the optimal dose that we previously determined (0.05 mg/mL) (Table 2) was used in the ahr-1(ju145) and ahr-1(ia3) mutant strains. Figure 4 shows that the increase in survival caused by PE was maintained in the ahr-1(ju145) strain (*p* < 0.0002), but it was lost in the ahr-1(ia3) mutant.

We previously mentioned that the ahr-1(ia3) strain presents a deletion that shortens the PAS domain. It could be hypothesized that the ahr-1(ju145) mutant maintains some activity, and the ahr-1 (ia3) mutant loses it completely. This may explain the result obtained, and thus, we conclude that the AHR-1 receptor mediates the positive effect of PE on the longevity of *C. elegans*.

### 2.4. Effect of PE on Motility in C. elegans

**Figure 5:**
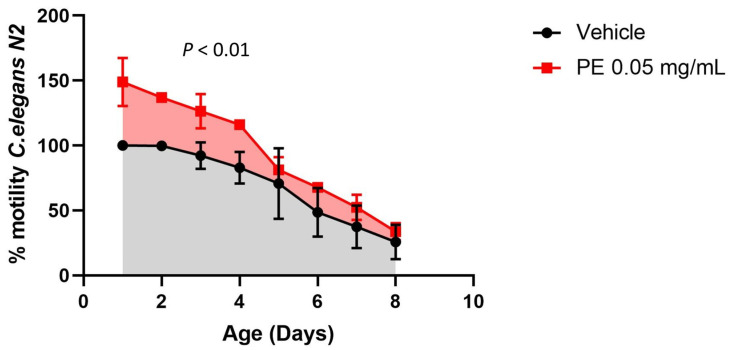
The effect of PE supplementation 0.05 mg/mL on *C elegans* motility at different ages. The 100% value is the motility of control worms (vehicle) at 1 day of the adult stage. PE: pomegranate extract. *p* < 0.01.

To evaluate the effect of PE on the vitality of the *C. elegans* worm, we created motility curves of the nematodes in each of the different doses of pomegranate extract PE (0.005, 0.01, 0.05, 0.1, 0.5, and 1 mg/mL) during the first eight days of the adult worm stage. In the first place, worms fed with doses of 0.1, 0.5, and 1 mg/mL did not present a positive effect on the motility of the worms compared with the population fed on control conditions. Subsequently, lower doses of this extract (0.005, 0.01, and 0.05 mg/mL) were analyzed. In this case, an increase in the motility of the worms supplemented with 0.05 mg/mL was observed (*p* < 0.01) (Figure 5). Doses of 0.005 and 0.01 mg/mL did not increase the motility of the worms.

### 2.5. Nutritional Intervention with Pomegranate Extract on AHR Pathway Gene Expression in Mouse Liver

**Figure 6:**
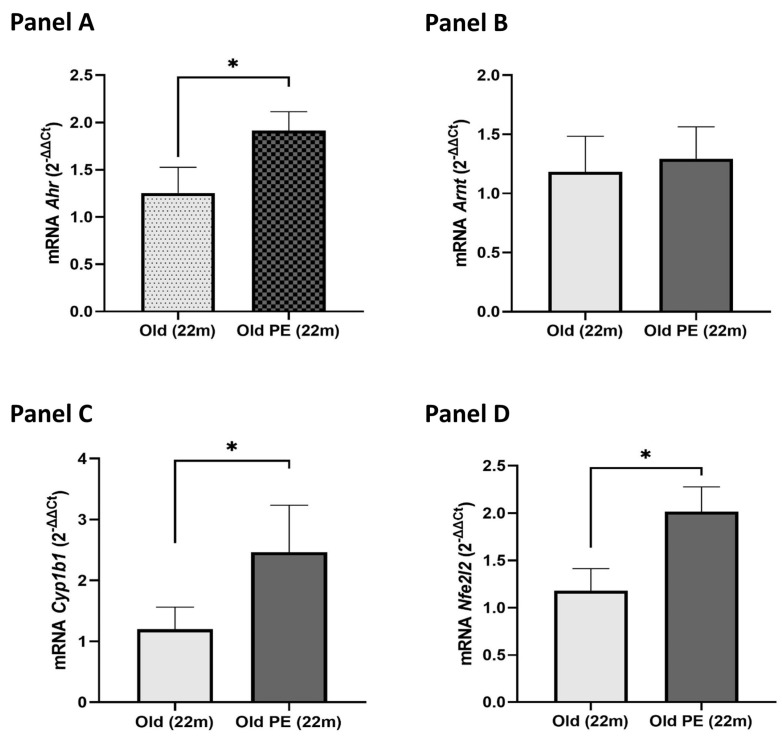
Effect of PE supplementation on expression of *Ahr* (**Panel A**), *Arnt* (**Panel B**), *Cyp1b1* (**Panel C**), and *Nfe2l2* (**Panel D**) in mouse liver. Results are expressed as mean ± SEM (*n* = 5–6 per group). * *p* < 0.05.

In our mouse model, we aimed to determine whether pomegranate extract supplementation modulated the genes involved in the AHR pathway in the liver, where this pathway plays a significant role in the detoxification process. As shown in Figure 6, PE significantly affected the expression of nearly all the genes associated with the AHR pathway. Specifically, the expression levels of *Ahr*, *Cyp1b1*, and *Nfe2l2* (*Nrf2*) increased approximately two-fold in the liver of the supplemented group compared to the non-supplemented group at 22 months of age.

## 3. Discussion

In this work, we observed that the AHR pathway is maintained in centenarians (extraordinary aging), while it declines significantly in septuagenarians, representing ordinary aging. The AHR pathway plays a fundamental role not only in xenobiotic detoxication but also in many physiological functions related to the immune system, the homeostasis of carbohydrate metabolism, and other functions [1]. The results of expression in centenarians led us to analyze the implication of this pathway in longevity using *C elegans* as an animal model widely used in aging studies [21].

Pomegranate has been considered as a “super fruit” due to the numerous beneficial properties for health [22]. Zheng and co-workers observed that pomegranate juice and extract extend the lifespan in *C. elegans* but did not provide a mechanistic explanation [23]. Moreover, Kılıçgün and co-workers proposed that pomegranate could be considered as a supplement to enhance longevity and fertility [24].

In this work, we suggest that the activation of AHR could be one of the key mechanisms involved in the increased longevity associated with PE administration.

Eckers et al. observed that ahr-1(ju145) mutants had a longer mean lifespan in *C. elegans* [20]. Thus, their results are contradictory with ours. This may be due to different technical conditions [20]. They used the *E. coli* HT115 strain as food for worms. Survival was determined visually, and they changed the culture medium every three days. We used the *E. coli* OP50 strain as food, FUdR was used to avoid progeny without medium refresh, and the survival was analyzed using an automated system based on artificial vision. Indeed, to determine if these methodological differences could be responsible for the observed discrepancy, our experiments were repeated using the bacterium *E. coli* HT115 or *E. coli* OP50 as a food source, transferring the worms to fresh NGM plates three times a week and analyzing the survival accordingly. A reduction in the survival of the mutants compared to the wild-type strain was observed under these conditions. 

Thus, our results show that mutations of AHR-1 in *C. elegans* have a negative effect on lifespan, and this is reversed with PE supplementation in worms in which the sequence involved in ligand recognition is not affected (PAS domain). However, we did not observe changes in longevity when we used worms displaying a deletion in the regulatory moiety. Therefore, we confirm that the AHR-1 receptor mediates the positive effect of PE on the longevity of *C. elegans*. Our findings indicate that PE supplementation not only extends lifespan but also enhances vitality in worms. Additionally, we observed in mice that PE modulates the AHR pathway in the liver, a vital organ in the detoxification process where AHR plays a crucial role.

Pomegranate peels are a by-product in the processing of pomegranate products, which are usually discarded as a waste. However, a large number of studies has shown that pomegranate peel extract is rich in a variety of phenolic substances. Ellagic acid is one of the main active components that has significant biological activities, such as its antioxidant, anti-tumor, anti-inflammatory, neuroprotective, anti-viral, and anti-bacterial activities [25,26].

Urolithins are a family of compounds derived from ellagic acid that are generated in vivo from bacterial transformations of the colonic microbiota. They were identified as potential biomarkers of the consumption of ellagitannin-rich foods such as pomegranate in humans [27,28]. Urolithins (urolithin A, urolithin B, and isourolithin A) are detected in high amounts in the colon [29] and are subsequently absorbed, so they can be found in the blood circulation and in tissues such as the prostate, breast, etc., for several days after pomegranate consumption [30,31,32]. Supplementation with our PE results in detectable plasma levels of urolithin A in mice [33]. Thus, we propose that PE displays favorable effects on life- and healthspan in *C. elegans*. These findings suggest that nutritional interventions like PE supplementation could promote healthy aging in humans, potentially through a beneficial enhancement in the AHR pathway.

### Limitations of this Study

Our findings suggest a potential role for the AHR pathway in influencing lifespan, particularly in the context of extreme longevity observed in centenarians. However, the current study mainly used worms with loss-of-function mutants to investigate the effects of reduced AHR-1 expression on lifespan. While these approaches provide valuable information, they do not directly address whether AHR overexpression can extend lifespan, which is crucial to fully understand the pathway’s role in longevity. To validate and extend our findings, future research should incorporate overexpression models that more closely mimic the elevated AHR expression observed in centenarians.

## 4. Materials and Methods

### 4.1. Human Study

#### 4.1.1. Participants

The Spanish Centenarian Study Group at RETICEF began in 2007 as a population-based study of all centenarians living within an area near of Valencia called La Ribera (11th Health Department of the Valencian Community, Spain), which comprises 29 towns (240,000 inhabitants). Potential subjects were selected from the population data system of the 11th Health Department. For this study, we used 9 centenarians (C), 8 septuagenarians with family-linked centenarians (centenarian’s offspring) (D), and 10 septuagenarians (S) (non-centenarian offspring) [34]. All individuals or their relatives were informed about the aims and scope of this study, including all potential risks. Written informed consent was obtained before participation. The inclusion criteria were as follows: (i) centenarians (C), being aged 97 or older at the beginning of this study (20); (ii) D, centenarian offspring: community-dwelling individuals aged 65–80 years old having a mother or father who lived to or beyond the age of 97 years; and (iii) septuagenarians or non-centenarian offspring, being age (±5 years) and collecting data on sex and place of birth and residence and having matched controls for D (1:1) with non-long-lived parents (i.e., deceased before 90 years). Furthermore, all individuals had to be habitual residents (more than 6 months a year) in the health department of La Ribera (Valencia, Spain) and be included in the population database. Having a terminal illness or a life expectancy of less than 6 months by any cause was considered an exclusion criterion for all groups. All experimental procedures were performed following the principles of the Declaration of Helsinki of the World Medical Association and according to the national and international guidelines. The study protocol was approved by the Committee for Ethics in Clinical Research of the Hospital de la Ribera, Alzira, Spain.

#### 4.1.2. Peripheral Blood Mononuclear Cell Isolation

Whole blood was obtained from each subject using VACUTAINER^®^ CPT™ tubes (BD, Franklin Lakes, NJ, USA) containing sodium heparin as an anticoagulant. Within 0.5 h of collection, blood was processed on-site according to the manufacturer’s instructions. This involved centrifugation at 3000× *g* at room temperature for 15 min. Following centrifugation, the cell preparation tubes were gently inverted to facilitate the separation of plasma, mononuclear cells, and erythrocytes. The white ring containing mononuclear cells was carefully collected. Subsequently, mononuclear cells were washed twice in PBS and stored at –80 °C for RNA isolation at a later stage.

#### 4.1.3. Isolation of Total RNA from Peripheral Blood Mononuclear Cells

Total RNA was isolated using a mirVana miRNA Isolation Kit (Ambion, Austin, TX, USA) following the manufacturer’s protocol. The purity and concentration of RNA were assessed using OD260/280 readings obtained with a Genequant Pro Classic spectrophotometer (GE Healthcare Biosciences, German). RNA integrity was determined by capillary electrophoresis using the RNA 6000 Nano Lab-on-a-Chip kit and Bioanalyzer 2100 (Agilent Technologies, Santa Clara, CA, USA). 

#### 4.1.4. Gene Expression Profiling and Microarray Data Analysis

mRNA profiling was performed using Clariom D-Human Array (Thermo Fisher Scientific, Madrid, Spain), which comprises >542,000 transcripts constituting over 134,000 gene-level probe sets. Microarray experiments were conducted according to the manufacturer’s instructions (Thermo Fisher Scientific). Specifically, 150 ng total RNA was labeled using the GeneChip WT Plus Reagent Kit. The labeling reaction was hybridized on the array using a Hybridization Oven 645 at 45 °C for 16 h. The arrays were stained with Fluidics Station 450 using fluidics script FS450_0001 and scanned on GeneChip Scanner 3000 7G. GeneChip Command Console software v4.0 supplied by Thermo Fisher Scientific was used for gene expression analysis.

Data (. CEL files) were analyzed and statistically filtered using software Partek Genomic Suite 6.6 (Partek Inc., St. Louis, MO, USA). Input files were normalized with the RMA algorithm for gene arrays on core metaprobesets. A 1-way ANOVA was performed with the Partek Genomics Suite across all samples. Statistically significant genes between different groups were identified using a model analysis of variance with *p* ≤0.05. In this work, our focus was on genes integrated into the AHR pathway to examine their expression changes in the three study groups. 

### 4.2. C. elegans Study

The *C. elegans* strains used were wild-type strain N2 and CZ2485 ahr-1(ju145) and ZG24 ahr-1(ia3) mutants, both obtained from the Caenorhabditis Genetics Center collection.

The natural concentrated extract of whole pomegranate (Pomanox^®^ P30), obtained through a water-based extraction process, was provided by Euromed (Barcelona, Spain). The analysis of the Pomanox^®^ batch was carried out in the Euromed Quality Control laboratory. For this, the HPLC-UV liquid chromatography technique was used, with a diode array UV detector (Diode Array Detector, DAD), and the polyphenols α-punicalin and β-punicalin, α-punicalagin and β-punicalagin, and ellagic acid were determined to certify the batch. According to the manufacturer, Pomanox^®^P30 (Batch No 0100531601) was standardized to >60% total (poly)phenols and punicalagins α + β ≥ 30% p/p and contains α-punicalin and β-punicalin (0.592 g/100 g and 0.648 g/100 g, respectively), α-punicalagin and β-punicalagin (13.311 g/100 g and 17.255 g/100 g, respectively), and ellagic acid (2.470 g/100 g).

The PE was dissolved in water and added to the surface of the *C. elegans* culture medium (NGM: nematode growth medium) plates. The extract was evaluated at different doses, depending on the functionality analyzed (lifespan or motility tests). 

#### 4.2.1. Lifespan Study

To study the effect of PE on the lifespan of *C. elegans*, worms of the *C. elegans* N2 strain or ahr-1(ju145)I (CZ2485)/ahr-1(ia3)I (ZG24) mutants were obtained, synchronized in age, and cultured in NGM and NGM supplemented with the different doses of PE (0.005, 0.01, 0.05, 0.1, 0.5, 1, 5, 10 mg/mL). The bacterium *E. coli* OP50 was added as a food source. FUdR was used to avoid progeny. The survival of each condition was detected by an automated system based on artificial vision developed by the ADM Biopolis company. Thus, survival curves were obtained for each condition used.

To reproduce previously published conditions [20] with the ahr-1(ju145)I (CZ2485)3 strain, survival assays were performed by transferring the worms to fresh NGM plates three times per week and analyzing manually. The bacterium *E. coli* HT115 was added as a food source. The statistical analysis of the survival curves was performed using the log Rank T test with the GraphPad Prism 9 software (GraphPad software, San Diego, CA, USA).

#### 4.2.2. Assessment of Vitality in *C. elegans*

The effect of PE on vitality was evaluated by determining the motility of *C. elegans*. For this, an automated system based on artificial vision was used. This system makes it possible to detect the activity of the worms during their adult stage. For this, worms of the wild strain N2 of *C. elegans* were cultured in 96-well plates containing standard culture (NGM) or NGM supplemented with each of the different doses (0.005, 0.01, 0.05, 0.1, 0.5, and 1 mg/mL). The motility of the nematodes was measured daily for eight days. To normalize the data from independent experiments, the results were represented as the percentage of motility of the treated worms compared with controls. The experiments were performed in duplicate.

To compare the motility of each condition compared with the control on a specific measurement day, a t-test statistical analysis was applied using the GraphPad Prism 9 software.

### 4.3. Mouse Study

#### 4.3.1. Experimental Animals

This animal study was approved by the University of Valencia Ethics Committee for Research and Animal Welfare (license reference: A20201110122413). Animals were housed at the Animal House Core Facility of the University of Valencia.

Male C57BL6 (wild-type, WT) eighteen-month-old mice were assigned to two groups: a control group (only drinking water) and another PE supplementation (150 mg/kg/day) group (solubilized in water).

The PE dosage was adjusted to the liquid intake and to the animals’ body weights every 3 days during 16 weeks’ supplementation. The dose was chosen based on the positive effects found both in metabolic and cardiovascular function in previous clinical studies. As PE was dissolved properly in water, it was used as a vehicle in the control group.

#### 4.3.2. Total RNA Extraction and Real-Time Polymerase Chain Reaction (RT-PCR) Studies

Liver tissue was extracted using 300 μL of TRIzol reagent. Integrity quality was analyzed by Nanodrop 2000 (Agilent, Santa Clara, CA, USA), and the purity was evaluated with the 260/280 ratio. RT-PCR was performed for each of the following genes, using ready-to-use primer and probe sets pre-developed by Applied Biosystems (Foster City, CA, USA, TaqMan Gene Expression Assays) using Quantum Studio v5 (QuantStudioTM Design & Analysis Software v1.4.2), establishing the proper conditions. The housekeeping gene was *Gapdh* (Mm99999915_g1, Applied Biosystems), and the genes studied were *Ahr* (Mm00478932_m1, Applied Biosystems), *Arnt* (Mm00507836_m1, Applied Biosystems), *Cyp1b1* (Mm00487229_m1, Applied Biosystems), and *Nfe2l2* (*Nrf2*) (Mm00977784_m1, Applied Biosystems). For the analysis of the results, 2^−∆∆Ct^ was used.

#### 4.3.3. Statistical Methods

Values are expressed as the mean ± standard deviation (SD) or mean ± standard error (SEM). The normal distribution of the samples was assessed using the Shapiro–Wilk test. To compare two different groups, the unpaired Student’s *t*-test was used or the Mann–Whitney test in the case of a non-normal distribution. Statistical analysis was performed using Statistical or GraphPad Prism software with the significance level set at *p* < 0.05, and all graphs were created with GraphPad Prism8 Software version 9.0.0.

## Figures and Tables

**Table 1:** Fold changes in mRNA expression genes of AHR signaling pathway.

	*AHR*	*ARNT*	*CYP1B1*	*NFR2*
**C vs. S**	2.02 (*p* = 0.003)	1.34 (*p* = 0.03)	1.69 (*p* = 0.03)	1.68 (*p* = 0.002)
**C vs. D**	1.67 (*p* = 0.03)	1.08	1.43	1.09
**D vs. S**	1.20	1.24	1.18	1.55 (*p* = 0.01)

C: centenarians; D: centenarian’s offspring; S: septuagenarians. In values that are non-statistically significant, *p* > 0.05, no *p* values are shown.

**Table 2:** Effect of PE on lifespan in *C. elegans*.

PE Doses (mg/mL)	Positive Effect on Lifespan	Increment Half-Life vs. Vehicle (Days)	Increment Maximal Lifespan vs. Vehicle (Days)
0.005	*p* > 0.05	0	0
0.01	*p* > 0.05	0	0
0.05	*p* < 0.0001	6	2
0.1	*p* < 0.0001	2	1
0.5	*p* < 0.001	2	0

Half-life: day that population has 50% mortality. Maximal lifespan: day that population has 100% mortality. Vehicle: control condition or non-supplemented group. PE: pomegranate extract.

## Data Availability

Data are contained within this article.
